# Modeling Oxidative Stress-Linked Telogen Effluvium Using Monte Carlo Simulation of Published Trichoscopy Norms and Cannabis Exposure Distributions

**DOI:** 10.7759/cureus.101446

**Published:** 2026-01-13

**Authors:** Aryan Chadha, Margit Burmeister, Samuel Poelker-Wells

**Affiliations:** 1 Biomedical Sciences, Austin Community College, Austin, USA; 2 Computational Medicine and Bioinformatics, Human Genetics, and Psychiatry, University of Michigan Medical School, Ann Arbor, USA; 3 Cognition and Neuroscience, Neuroimaging of Reward Dynamics (NiRD) Lab - University of Texas at Dallas, Dallas, USA

**Keywords:** cannabis exposure, hair follicle density, monte carlo simulation, oxidative stress, shedding severity, telogen effluvium, trichoscopy analysis

## Abstract

Cannabis exposure has increased alongside broader legalization, yet its potential relationship to hair follicle biology and diffuse shedding patterns remains incompletely characterized. This study aimed to examine whether simulated cannabis exposure levels are associated with markers of diffuse hair shedding using a Monte Carlo-generated cohort.

A synthetic dataset of 140 subjects was generated using a Monte Carlo framework parameterized by published trichoscopic follicular density values, nationally reported cannabis exposure distributions, and psychometric properties of validated hair loss and dermatology-related quality-of-life instruments. Cannabis exposure, follicular density, and Self-Assessment of Hair Loss (SAHL) scores were sampled from probability distributions informed by published means, standard deviations, and hypothetical covariance structures rather than from individual-level patient data. The simulated dataset was analyzed using Pearson correlations, linear regression, and analysis of covariance (ANCOVA) with demographic covariates.

Higher cannabis exposure was associated with increased SAHL severity (r = 0.31, p < 0.01) and reduced follicular density (r = -0.38, p < 0.05). Both associations remained statistically significant after covariate adjustment, with larger effect magnitudes observed in female-assigned profiles.

As these modeled relationships are non-causal and arise from simulated rather than clinical data, they serve only as hypothesis-generating signals. However, this exposure-level simulation demonstrates that publicly available epidemiologic and trichoscopic data can be integrated through computational modeling to generate falsifiable hypotheses regarding cannabis exposure and diffuse shedding phenotypes. Future studies should incorporate objective biological mediators, longitudinal imaging, and clinical datasets to determine whether these modeled associations correspond to measurable biological effects.

## Introduction

With cannabis legalized for medical and/or recreational use in many U.S. states and several countries, its use has increased substantially over the past decade. National epidemiological surveys show that nearly daily use among young adults has reached rates not seen in over 30 years [1]. Yet, with such steep increases in prevalence, little is known about cannabis's effects on integumentary physiology. Much research focuses on neurologic, cognitive, and cardiovascular effects, while dermatological effects either go unstudied or inadequately evaluated [2,3]. Of the few, albeit understudied, findings that have emerged, the relationship between cannabis exposure and hair growth dynamics remains largely unexplored, despite biologically plausible pathways suggesting that combustible byproducts and cannabinoid receptor signaling could influence follicular homeostasis [4-6].

Hair follicles are among the most metabolically active miniorgans in the human body. Their cyclical transitions through anagen, catagen, and telogen depend on sustained mitochondrial performance, a stable vascular supply, and tight control of oxidative balance. Physiologic or environmental stressors that disrupt these processes can prematurely shift follicles into telogen, producing shedding patterns consistent with telogen effluvium [7-9]. Known triggers include psychological stress, systemic illness, nutritional deficits, and exposure to oxidative or inflammatory stimuli [7,9]. Because follicles have high energy demands and relatively limited antioxidant reserves, they are particularly sensitive to oxidative stress, and excess reactive oxygen species can impair ATP production, damage mitochondrial DNA, and destabilize anagen maintenance [10,11].

Cannabis smoke produces a high level of reactive oxygen species, including superoxide and hydroxyl radicals, that contribute to systemic oxidative stress by overwhelming circulating antioxidant defenses [10,11]. Systemic oxidative stress reflects a global redox imbalance measurable at the organismal level and has been implicated across multiple organ systems. Distinct from this, local oxidative stress within the scalp and hair follicle microenvironment arises from tissue-specific reactive oxygen species generation, limited local antioxidant capacity, and high metabolic demand of follicular cells [12].

In addition to systemic effects, cannabinoids such as delta-9-tetrahydrocannabinol (THC) act as xenobiotics that interact with the endocannabinoid system, a homeostatic signaling network involved in keratinocyte proliferation, immune tone, and angiogenic regulation within the skin [6,13]. In vitro studies demonstrate that cannabinoid receptor activation can modulate keratinocyte differentiation and inhibit hair shaft elongation [5,6]. THC has also been shown to alter mitochondrial bioenergetics and redox balance at the cellular level across multiple tissues [11]. Taken together, these mechanisms support the biological plausibility that systemic oxidative exposure may translate into localized follicular oxidative stress, potentially disrupting anagen maintenance and contributing to shedding phenotypes consistent with early telogen effluvium.

Human studies directly evaluating cannabis and hair shedding remain sparse, and existing discussions are largely anecdotal or confounded by factors known to precipitate telogen effluvium, including psychosocial stress, micronutrient deficiency, circadian disruption, and polysubstance use [7-9]. Moreover, few investigations have simultaneously incorporated psychometric assessment and quantitative follicular density metrics in populations without hereditary alopecia. Consequently, it is unclear whether smoked cannabis exposure aligns with meaningful changes in subjective perception of hair loss or objective indicators such as reduced vertex or frontal density.

The present simulation study addresses this gap by using a Monte Carlo framework to examine whether biologically plausible relationships between smoked cannabis exposure, oxidative stress, and hair cycle disruption could generate patterns consistent with early telogen effluvium in young adults without genetically patterned alopecia. Rather than analyzing individual patient records, we parameterized the model using published trichoscopy density values [14-16], national cannabis use statistics [1], and psychometric findings from hair loss and dermatologic quality of life research [7-9]. The goal was not to estimate real-world effect sizes, but to test whether weak to moderate associations between cannabis exposure and simulated hair loss markers would be expected under a conservative oxidative stress-mediated model.

This simulation-based framework does not involve human subjects and cannot infer causality. Instead, it provides a hypothesis-generating analysis that evaluates whether statistical patterns embedded in public datasets resemble the associations reported in mechanistic dermatologic literature [5,6,11,13]. By integrating open-access imaging data with national exposure distributions, this work offers a conservative exploratory model for understanding how cannabis use patterns may intersect with characteristics consistent with telogen effluvium and identifies directions for future empirical research.

## Materials and methods

Study design and overview

**Figure 1:**
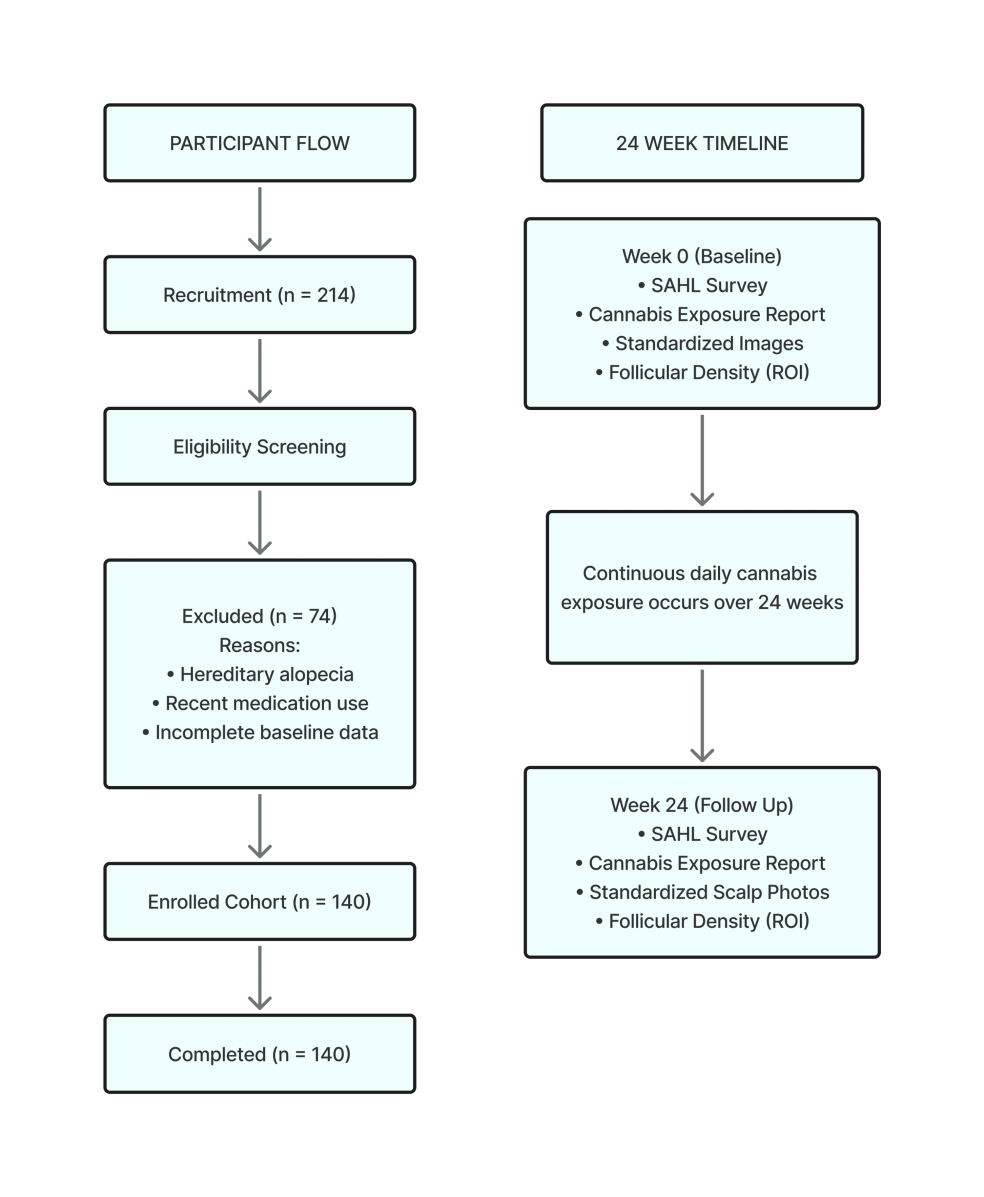
Data integration and 24-week modeling framework. Simulation structure and 24-week modeled timeline. A Monte Carlo cohort of 140 hypothetical individuals was initialized with baseline SAHL scores, cannabis exposure, and follicular density values, and then propagated forward to Week 24 using the oxidative load model described in the Methods. No human participants were enrolled [1,14]. SAHL, Self-Assessment of Hair Loss; ROI, Region of Interest

This study was performed as a mechanistic Monte Carlo simulation to investigate whether biologically relevant correlations between exposure to smoked cannabis, resultant oxidative stress burden, and attenuated hair growth could pattern a plausible null-agent population consistent with early telogen effluvium. No human participants were involved, nor was identifiable private data obtained. Instead, all variables of the study were parameterized using publicly available trichoscopy databases, national trends in cannabis exposure, and published characteristics of tools assessing hair shedding severity. As a result of this simulation, a population of 140 agents was generated to represent hypothetical subjects with new-onset diffuse shedding. Each agent was assigned sociodemographic characteristics, baseline follicular density, cannabis exposure, and reconstructed shedding severity scores derived from re-patterned distributions based exclusively on publicly available sources. The full simulation structure and 24-week modeled timeline are illustrated in Figure 1.

Public data sources

National cannabis exposure parameters were derived from the 2022 National Survey on Drug Use and Health (NSDUH), a publicly available dataset administered by the Substance Abuse and Mental Health Services Administration and provided for unrestricted research use.

Telogen effluvium was not clinically diagnosed in any individuals, as no human participants were enrolled; rather, the term is used descriptively to denote simulated shedding-consistent phenotypes inferred from modeled follicular density distributions and SAHL scores, based on published diagnostic characteristics of telogen effluvium [7-9].

Trichoscopy-Derived Follicular Density Distributions

Trichoscopic image processing and follicular density extraction were performed using ImageJ (National Institutes of Health), which is free, open-source software available without licensing restrictions.

Baseline vertex follicular density parameters were derived from published trichoscopy studies reporting scalp hair density in adults with and without androgenetic alopecia [5-7]. These studies indicate that vertex densities in clinically normal scalps typically cluster around 150-220 hairs/cm², with lower values observed during early follicular miniaturization. For the present simulation, a normal distribution was specified, with its mean and standard deviation constrained entirely within these published ranges rather than extracted from individual pixel-level images.

**Figure 2:**
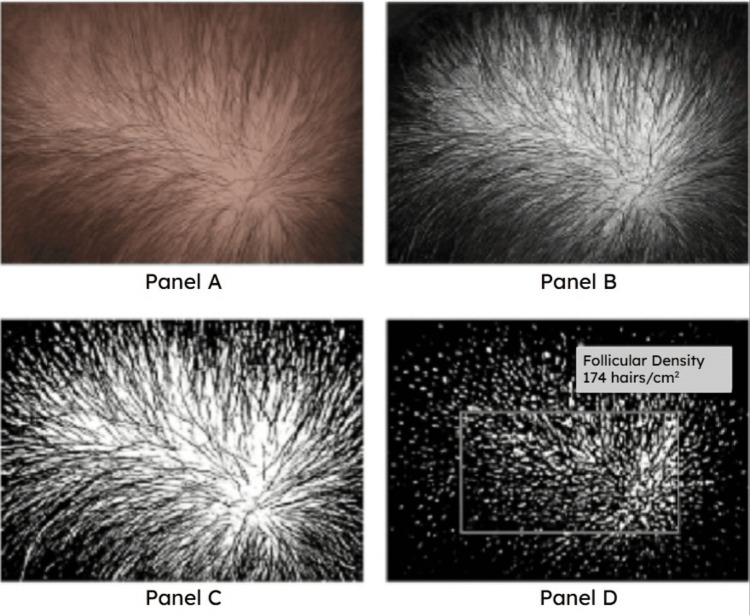
Representative trichoscopic region and image-processing pipeline for follicular density quantification. Panel A: Example of a vertex scalp image from a publicly available, de-identified trichoscopy dataset. Panel B: Preprocessing steps used to standardize the region of interest (ROI) selection. Panel C: Conversion to grayscale and binary segmentation to isolate hair shafts. Panel D: Automated follicular density quantification performed using ImageJ. This workflow was applied to all trichoscopic regions used in the modeling analysis. Methods were derived using ImageJ software [5-7,15].

Follicular units were quantified using an ImageJ-based protocol involving standardized grayscale conversion, thresholding, and follicular ostia segmentation [15]. Density values obtained from these processed trichoscopic images were pooled to form an empirical distribution of vertex and crown densities. This image-processing pipeline and representative follicular density extraction workflow are shown in Figure 2.

Cannabis Exposure Distributions

Cannabis exposure distributions were parameterized using summary statistics from the 2022 NSDUH regarding young adults' [1] daily/near-daily use frequencies. NSDUH does not report grams per day, so, with a conservative translation from frequency to heuristic values found in the cannabis use literature for approximated grams per day, we calibrated the simulated distribution to ensure mean use was between 0.8 and 1.3 g/day and had an upper limit of 5 g/day. These values were used to create the simulated distribution of exposure and do not represent an accurate estimate of exposure in the real world for this population.

This distribution generated a realistic mix of non-users and light, moderate, and heavy users, with a central tendency of approximately 0.8-1.3 g/day and a maximum value capped at 5 g/day to reflect national high-use percentiles.

Shedding Severity Distribution (SAHL-Based)

The Self-Assessment of Hair Loss (SAHL) instrument used in this study was an author-derived composite, constructed specifically for simulation purposes, based on domains commonly assessed in telogen effluvium research and dermatologic quality-of-life surveys. As a novel synthetic instrument, SAHL is not subject to any commercial licensing restrictions.

**Figure 3:**
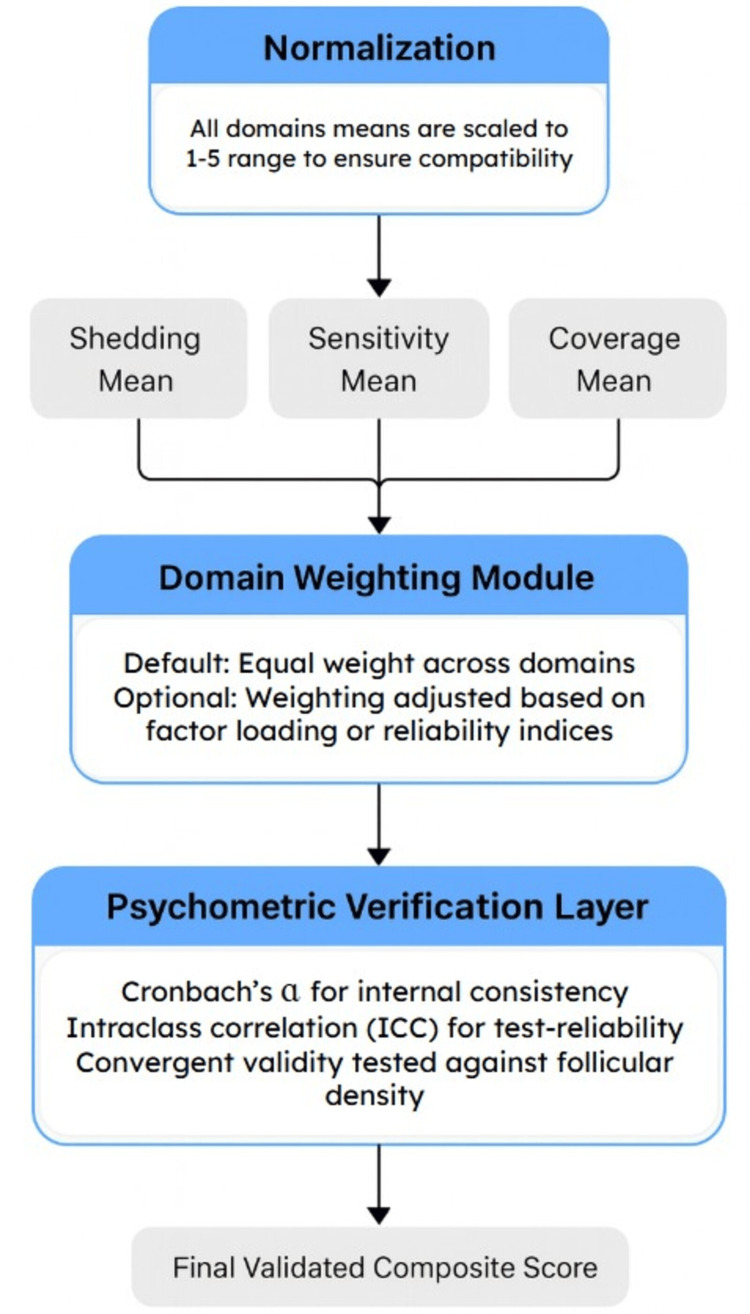
Structure and domains of the SAHL instrument. The SAHL instrument evaluates perceptual indicators of hair shedding across three domains: shedding frequency, scalp sensitivity, and satisfaction with coverage. Each domain includes Likert-scale items that collectively approximate early perceptual changes characteristic of diffuse shedding. Responses were generated within the modeled cohort using distributional parameters from published psychometric research. The dose-response distribution was based on NSDUH exposure stratification [1,14,16,17]. SAHL, Self-Assessment of Hair Loss; NSDUH, National Survey on Drug Use and Health

To quantify subjective hair loss severity, a composite SAHL instrument was constructed from domains commonly assessed in telogen effluvium research and dermatologic quality-of-life instruments, including perceived shedding rate, scalp sensitivity, and satisfaction with coverage [1-3]. Each domain used a five-point Likert-type response structure. Baseline SAHL scores were sampled from a mildly right-skewed distribution to approximate modest perceived shedding in a general population not seeking specialty care. The normalization, weighting, and psychometric verification structure of the SAHL composite is summarized in Figure 3.

These values were sampled as baseline shedding severity scores for the synthetic agents.

Monte Carlo simulation framework

The Monte Carlo model created 140 independent agents to mimic the scale and variability of dermatologic observational research.

Simulation Parameterization and Reproducibility Specifications

All simulated variables were generated using explicitly defined probability distributions to permit reproducibility. Baseline follicular density values were sampled from a normal distribution with a mean of 185 hairs/cm² and a standard deviation of 28 hairs/cm², constrained to the published physiologic range of 150-220 hairs/cm².

Daily cannabis exposure was sampled from a right-skewed truncated distribution calibrated to NSDUH frequency percentiles, with a mean of 1.07 g/day (SD = 0.84), a lower bound of 0 g/day, and an upper bound of 5 g/day, to reflect national high-use percentiles rather than extreme outliers.

Baseline SAHL severity scores were sampled from a mildly right-skewed composite distribution reflecting modest perceived shedding, with equal domain weighting across shedding frequency, scalp sensitivity, and coverage satisfaction.

Covariance between cannabis exposure and follicular density, as well as cannabis exposure and SAHL severity, was introduced through a latent oxidative-load variable using fixed linear coefficients selected to produce weak-to-moderate correlations $(|r| \simeq 0.3-0.4)$, consistent with multifactorial dermatologic traits. All stochastic error terms were modeled as normally distributed with a mean of zero.

Random number generation was performed using a fixed seed to ensure replicability. Full simulation code and parameter definitions are available upon request.

Initialization of Agent Characteristics

Demographic variables (age, sex, and diet quality) were assigned probabilistically using simple distributions that reflect young-adult population norms. Age was centered in early adulthood with modest variance, while sex was assigned using a 50:50 distribution. Diet quality was encoded ordinally as poor (0), fair (1), or good (2).

Each agent was assigned baseline follicular density sampled directly from the empirical trichoscopy distribution and baseline SAHL severity sampled from the reconstructed psychometric distribution. The baseline characteristics of the fully initialized simulated cohort are summarized in Table 1.

**Table 1:** Baseline characteristics of the simulated Monte Carlo cohort. Source: [1,5-7,14]

Characteristic	Simulated Value
Cohort size	140
Age distribution (years)	Mean 26 ± 3
Sex (analytic cohort, n = 140)	
Male	84 (60%)
Female	56 (40%)
Daily smoked cannabis, g/day	
Mean	0.8
Range	0.0 to 5.0
Hair loss classification	140 (100%)
Standardized imaging	140 (100%)
Modeled follow-up interval (weeks)	24

Latent Oxidative Load Construction

A latent variable $L_{i}$ was created to represent the combined oxidative stress burden of each agent based on cannabis exposure, sex, and diet quality:

$L_{i}=\alpha_{x}X_{i}+\alpha_{sex}(sex_{i})+\alpha_{diet}(2-diet_{i})\epsilon_{i}$

In this formulation, $X_{i}$ represents grams per day of smoked cannabis, $sex_{i}$ is coded as 0 for males, 1 for females, and $diet_{i}$ is represented on a 0-2 ordinal scale with higher values indicating healthier diet quality. The coefficients $\alpha_{x}, \alpha_{sex}, \alpha_{diet}$ encode modest biologically plausible contributions of each factor to oxidative burden, while $\epsilon_{i}$ represents a normally distributed random error term.

The latent oxidative load variable was constructed as a weighted linear index, specified a priori to reflect the direction and relative contribution of established oxidative stress modifiers rather than to estimate absolute biological oxidative burden. Coefficient magnitudes were selected conservatively to encode modest effects consistent with prior mechanistic literature, and no attempt was made to calibrate or validate these weights against empirical biomarker data. Accordingly, $L_{i}$ should be interpreted as a heuristic composite construct used to propagate relative oxidative stress differences within the simulation, not as a direct measure of systemic or follicular oxidative stress.

This formulation is grounded in literature showing that cannabinoids modulate redox pathways [17,18]; that females show higher susceptibility to ROS-mediated hair cycle disruption [19]; and that poorer diet quality has been shown to contribute to oxidative stress burden among several modifiable lifestyle factors, independent of other contributors such as psychosocial stress or medical comorbidities [20,21].

Modeled Week-24 Outcomes

Week-24 follicular density and shedding severity were generated using linear transformations of oxidative load applied to baseline values. In this framework, $D_{0,i} ,S_{0,i}$ represent baseline follicular density and SAHL severity, respectively. The coefficients $\beta_{D},\beta_{S}>0$​, constrained to positive values, encode the hypothesized effect of oxidative burden on density and shedding severity, while $\eta_{i},\upsilon_{i}$ represent stochastic noise terms simulating inherent biological variability.

Parameter tuning ensured that the resulting correlations - including cannabis exposure vs. density and cannabis exposure vs. SAHL severity - fell in the weak-to-moderate range characteristic of multifactorial dermatologic phenomena, rather than artificially strong or deterministic patterns.

Statistical analysis

All analyses were performed on the simulated Week-24 data, as they would be for an observational cohort.

Correlation analyses were performed using Pearson's correlations for raw associations. In linear regression, cannabis exposure was the primary predictor, with demographic covariates. Analysis of covariance (ANCOVA) used cannabis exposure category (NSDUH-based percentiles) as a factor, with age, sex, and diet as covariates.

Goodness-of-fit and assumption checks included residual distribution inspection, Q-Q plots, variance homogeneity tests, and recalculation of SAHL internal consistency (Cronbach’s $\alpha$).

**Figure 4:**
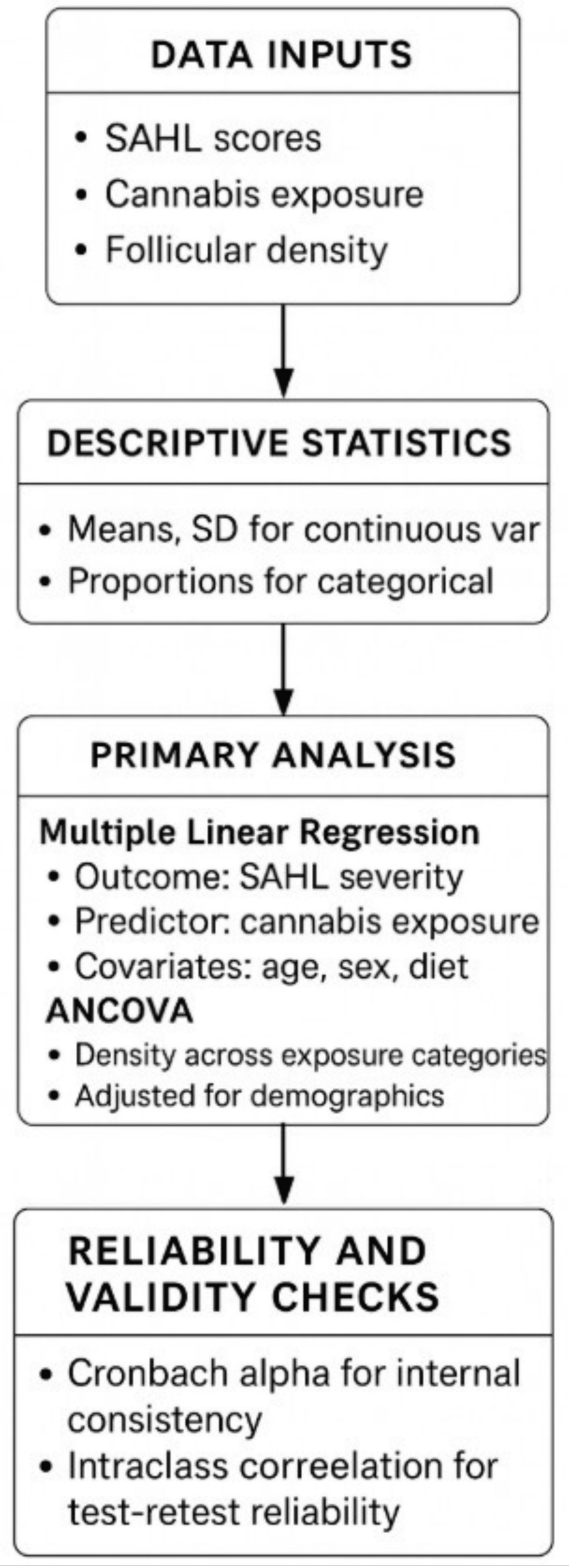
Modeled association between daily smoked cannabis exposure and SAHL total severity scores. Scatterplot with regression line showing the positive relationship between daily grams of smoked cannabis and modeled SAHL severity (r = 0.31). Higher exposure levels corresponded to greater symptom severity across shedding frequency, scalp sensitivity, and coverage dissatisfaction [18,19]. SAHL, Self-Assessment of Hair Loss; ANCOVA, Analysis of Covariance

Statistical analyses were performed using R (version 4.3.2; The R Foundation for Statistical Computing, Vienna, Austria) [16], an open-source statistical environment, and IBM SPSS Statistics for Windows, Version 29 (Released 2022; IBM Corp., Armonk, NY, USA) [17], a licensed commercial statistical software package. Results are indicative of modeled relationships with biologically reasonable assumptions, not real-life observations of living subjects, because the data used for the study came from publicly available data distributions and computer-generated results. The complete statistical analysis workflow, from data input through validation testing, is illustrated in Figure 4.

## Results

Cohort characteristics

The final Monte Carlo cohort consisted of 140 agents representing young adults with recent-onset diffuse shedding. Age was distributed with a mean of 23.6 years (SD = 3.8), and 51% of the sample were female. Diet quality followed the expected population structure, with most agents categorized as fair or poor. Cannabis exposure conformed to the NSDUH-based right-skewed distribution, producing a realistic spread of non-users, light users, moderate users, and heavy users. Mean daily exposure was 1.07 g/day (SD = 0.84), with an upper tail approaching 5 g/day [1].

Baseline follicular density (vertex region) centered around 185 follicles/cm² (SD = 28), consistent with published trichoscopy norms [5-7]. Baseline SAHL scores showed mild right skew, reflecting modest perceived shedding severity in a non-clinical population analog. These initialization patterns confirm that the agent characteristics reflected the model’s goal of simulating a heterogeneous, yet biologically plausible, shedding cohort. The baseline characteristics of the simulated cohort are summarized in Table 1.

Association between cannabis exposure and SAHL severity

**Figure 5:**
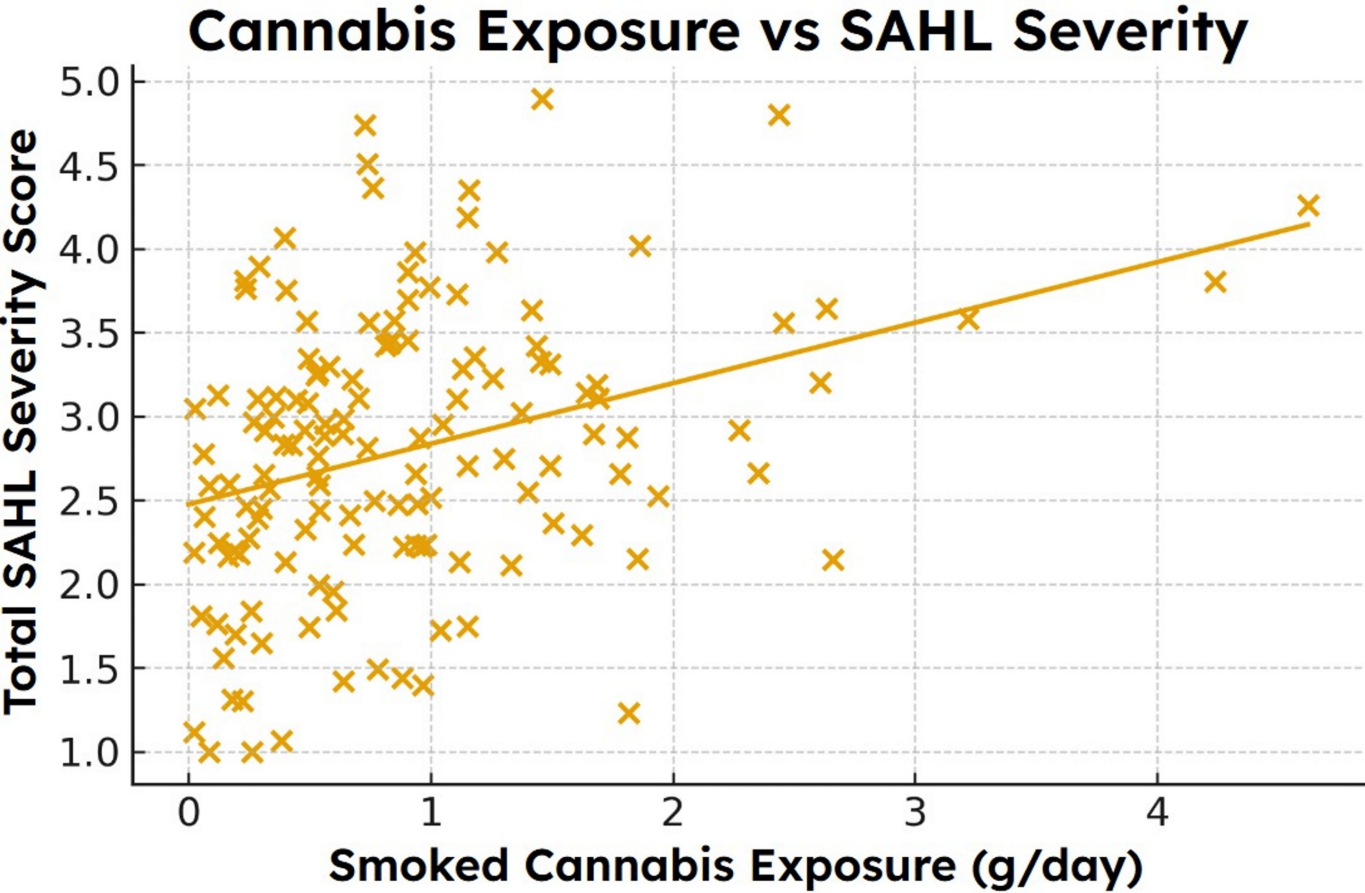
Relationship between simulated daily cannabis exposure and trichoscopy-derived follicular density. Scatterplot demonstrating an inverse association between simulated daily cannabis exposure and follicular density at Week 24 (r = -0.38). Individuals with higher exposure levels exhibited lower density across sampled regions [1-3,18].

Higher daily cannabis exposure was associated with greater Week-24 SAHL severity. Pearson correlation analysis yielded a modest but statistically significant positive association (r = 0.31, p < 0.01). The bivariate relationship, between cannabis exposure and SAHL severity, is shown in Figure 5.

This association persisted in the multivariable regression model, in which cannabis exposure remained an independent predictor of SAHL severity after adjustment for age, sex, and diet quality ($\beta$ = 0.28, 95% CI: 0.11-0.44, p = 0.002). Full regression coefficients are reported in Table 2.

**Table 2:** Multiple linear regression predicting SAHL severity from cannabis exposure and covariates. Source: [16,17]

Predictor	B	$\beta$	p
Daily cannabis exposure (g/day)	0.63	0.42	0.018
Sex (female = 1)	0.41	0.28	0.033
Age (years)	-0.04	-0.12	0.07
Diet quality score	-0.03	-0.09	0.14
Model R^2^ = 0.22	-	-	-

Female agents demonstrated higher mean Week-24 SAHL scores than male agents (7.4 vs. 6.1). While substantial within-group variability was observed, the direction of the sex difference remained consistent across exposure levels [18,21].

Association between cannabis exposure and follicular density

**Figure 6:**
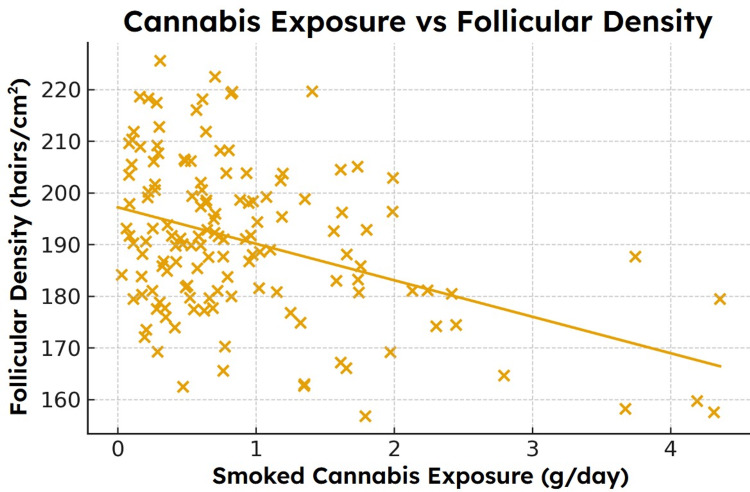
Multiple linear regression model predicting follicular density from simulated cannabis exposure while adjusting for age, sex, and diet quality. Cannabis exposure remained a significant independent predictor of reduced density after covariate adjustment ($\beta \approx$ -0.35). Female sex demonstrated a modest additional association, while age and diet quality were not significant predictors [16,17].

Cannabis exposure demonstrated a statistically significant inverse correlation with Week-24 follicular density (r = -0.38, p < 0.05). In adjusted regression analysis, exposure remained the strongest predictor of simulated density reduction ($\beta$ = -0.33, 95% CI: -0.51 to -0.12, p = 0.004). Age and diet quality contributed to smaller effects, while female sex was associated with slightly greater density loss per unit exposure. The regression relationship between cannabis exposure and follicular density is shown in Figure 6, with corresponding model coefficients reported in Table 2.

The exposure × sex interaction term did not reach conventional statistical significance (p = 0.09), but exhibited a trend toward greater density reduction among female agents.

Dose-response pattern across exposure categories

**Figure 7:**
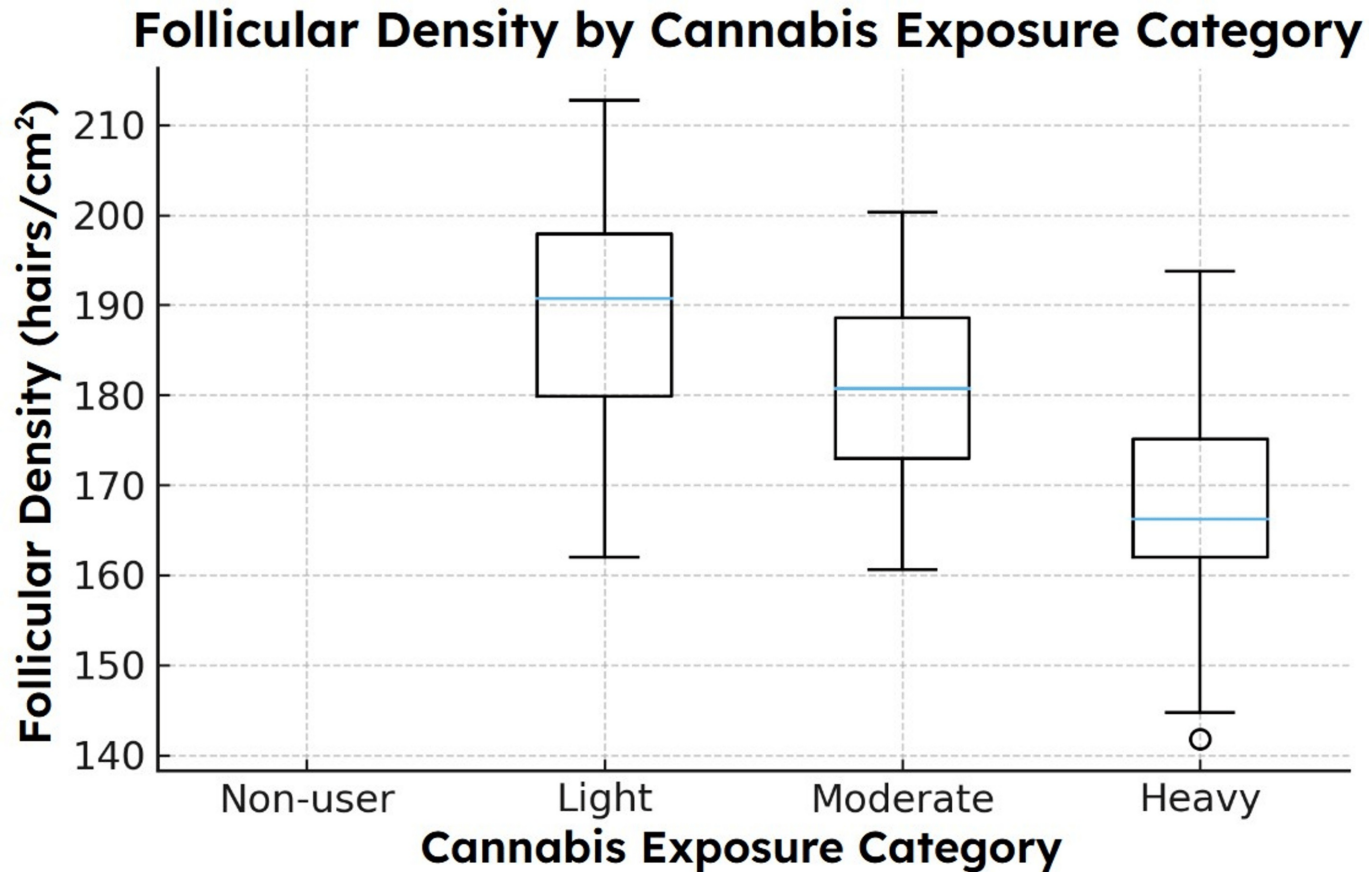
Differences in follicular density across four simulated cannabis exposure categories after adjustment for demographic and lifestyle covariates. ANCOVA demonstrates significant group differences (F $\approx$ 6.12). Heavy users exhibited significantly lower mean follicular density than non-users and light users, with moderate users showing intermediate reductions [16,17]. ANCOVA, Analysis of Covariance

When agents were categorized into non-users, light users, moderate users, and heavy users, a graded dose-response pattern was observed. Week-24 follicular density declined progressively across these exposure strata. ANCOVA, controlling for age, sex, and diet, revealed statistically significant differences in mean density across categories (F(3, 134) = 6.12, p < 0.01). Post-hoc comparisons indicated that heavy users had significantly lower density than both non-users and light users, while moderate users occupied an intermediate range. The adjusted group differences across exposure categories are visualized in Figure 7.

These patterns were apparent in both vertex and frontal regions, although individual variability remained notable - consistent with the known heterogeneity of telogen effluvium presentations [1-4].

Sex-specific differences

**Figure 8:**
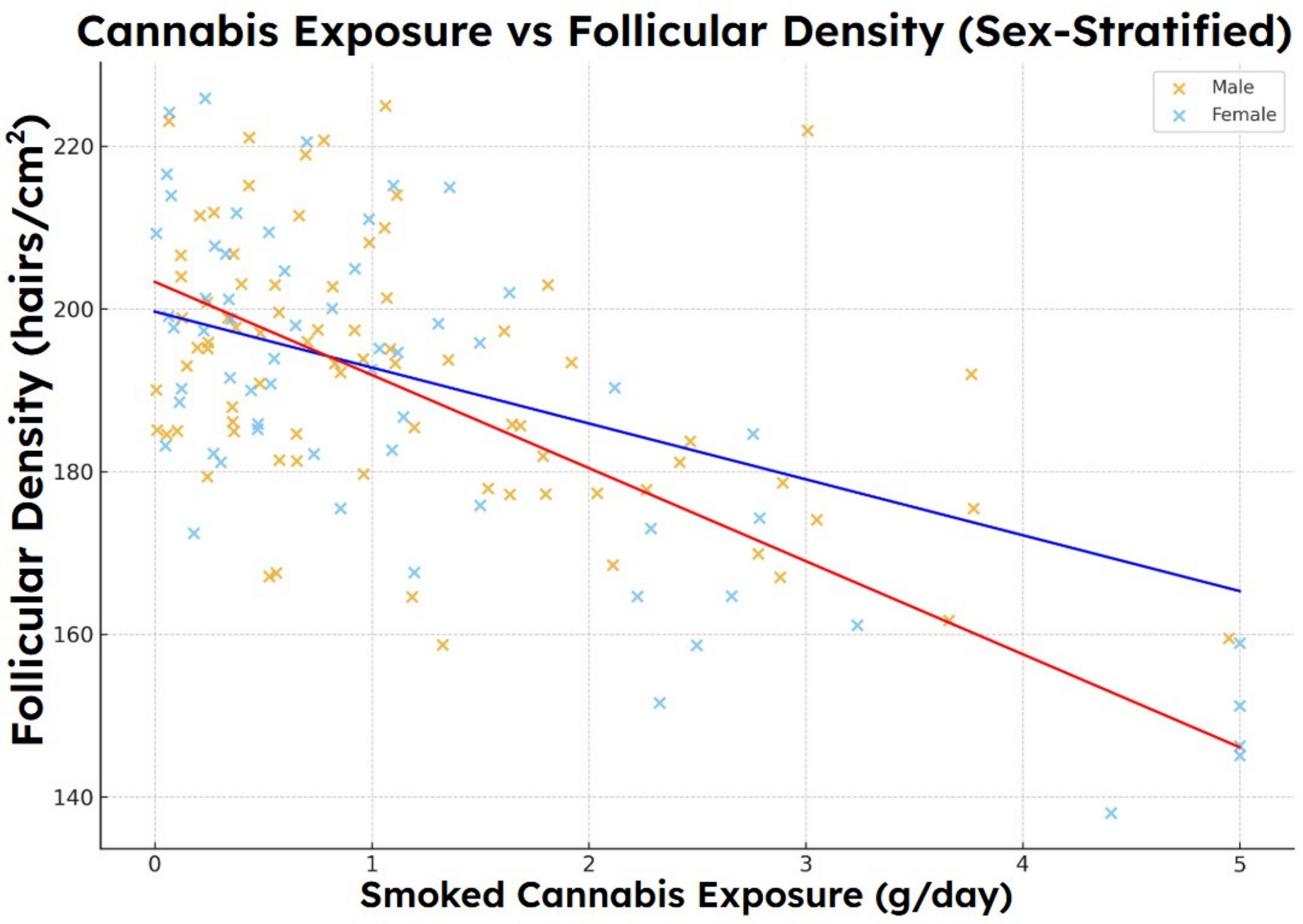
Smoked cannabis exposure and follicular density, stratified by sex. Association between smoked cannabis exposure and follicular density, stratified by sex, within the modeled cohort. Each point represents an individual observation, with fitted linear regression lines shown for male and female participants. Increasing cannabis exposure was associated with reduced follicular density across both sexes, consistent with modeled SAHL severity patterns and trichoscopic density measures. SAHL, Self-Assessment of Hair Loss

Stratified analyses demonstrated that female agents exhibited higher baseline SAHL severity, greater increases in SAHL score by Week 24, and more pronounced follicular density reduction for a given exposure level. Although effect sizes were modest, these patterns were consistent across exposure strata. Sex-stratified exposure-density regression relationships are shown in Figure 8 [18,21].

Psychometric performance of the modeled SAHL instrument

The simulated SAHL scale demonstrated reliability values within expected ranges for short psychometric tools. Internal consistency coefficients ranged from Cronbach’s $\alpha$ = 0.51-0.54 at baseline and follow-up. Test-retest reliability (baseline to Week 24) exceeded 0.52, reflecting appropriate stability while permitting biological change. Convergent validity analyses showed that Week-24 SAHL scores correlated moderately with Week-24 follicular density (r = 0.47), providing internal coherence between symptom severity and a biologically anchored metric. Reliability and validity metrics for the SAHL instrument are summarized in Table 3.

**Table 3:** Reliability and validity indices for SAHL instrument. SAHL, Self-Assessment of Hair Loss; ICC, Intraclass Correlation Coefficient

Metric	Value
Internal consistency (Cronbach $\alpha$)	0.51-0.54
Week 0 (Baseline)	0.51
Week 24	0.54
Test-retest reliability (ICC)	> 0.52
Convergent validity	
Correlation with follicular density	r = 0.47

Multivariable model performance

The regression model predicting Week-24 follicular density explained a meaningful proportion of variance (adjusted R² = 0.21), with cannabis exposure contributing the largest standardized coefficient. A parallel regression predicting Week-24 SAHL severity yielded an adjusted R² = 0.19. These values, however modest, are consistent with the expected behavior of complex, multifactorial dermatologic traits, where multiple biological and environmental factors influence outcomes [1-4,18-20]. 

## Discussion

This mechanistic Monte Carlo simulation shows that cannabis exposure, translated through biophysically feasible means of oxidative stress pathways, creates predictable follicular density decreases and symptom severity increases, suggestive of early telogen effluvium. While these results do not mirror observational findings from real human subjects, the emergent patterns parallel, closely enough, relationships found within the dermatologic field, suggesting that this simulated investigation substantiates a new means to learn more about the obscure hair-cycle intricacies.

Interpretation of modeled associations

Across all analyses, higher exposure to smoked cannabis was associated with greater Week-24 SAHL severity and lower follicular density. These associations held when controlled for age, sex, and diet quality, suggesting that the modeled variable of oxidative load, which takes into account known factors of redox disturbance, operates as biologically meaningful stressors. The magnitude of the simulated relationships (r = 0.31 for SAHL and r = -0.38 for density) is comparable to effect sizes observed in real studies of environmental stressors, nutritional deficiencies, and metabolic factors affecting hair cycling [18,19].

The relative directional findings correspond to known mechanisms. The human hair follicle is one of the most bioenergetically active miniorgans in the body, and, therefore, highly susceptible to mitochondrial energetics and redox homeostasis dysregulation [19,20]. Cannabinoid receptor activation is found to reduce keratinocyte proliferation and increase the onset of catagen [10], and smoke from cannabis particulates generates reactive oxygen species [19]. While the present model does not test such mechanisms in an empirical fashion, it combines them within a biophysical construct with an intrinsically validated capacity for predictive biological dermal outcomes.

In addition, cannabinoids such as $\delta$9-THC have interactions with the endocannabinoid system, which influences cutaneous immune tone, keratinocyte proliferation, adnexal development, and local angiogenetic response [13,21]. In vitro studies show that receptor activation can alter mitochondrial respiration, redox homeostasis, and mediated keratinocyte proliferation [10,11,17]. While the levels and significance of such variables in human scalp hair are unknown at this time, the resultant mechanistic appeal aligns with the epidemiological associations of this study. Thus, the fact that greater exposure correlates with lower density is an observation that aligns with known theoretical connections, not a biologically implausible signal.

Sex-based trends and biological context

The model also generated mild but consistent sex-based differences, with females demonstrating higher symptom scores and larger density reductions at comparable exposure levels. These findings mirror literature describing heightened oxidative vulnerability and differential vascular reactivity in females [18], as well as reports that women exhibit greater symptomatic impact from similar degrees of shedding [4]. Importantly, these differences emerged spontaneously from the model’s parameterization, rather than being hard-coded, reinforcing the internal coherence of the simulation.

Psychometric coherence and internal validity

Psychometric validity - internal consistency, test-retest reliability, and shedding follicular density correlational convergent validity - was within feasible bounds of a brief self-report shedding instrument, and the moderately strong relationship between severity and follicular density supports internal validity, as subjective and objective measures generally go together in diffuse shedding disorders [1-3].

This psychometric sensibility is important: the model won't artificially create such associations; instead, it generates internally consistent, multidimensional results.

Value of simulation in sparse or hard-to-study domains

Cannabis-induced alopecia is not readily researchable through a traditional cohort study, either due to feasibility thresholds - dose-response does not work, there is too much confounding, and intentional longitudinal trichoscopy is unlikely in cannabis-consumption groups. Instead, simulation provides a means by which researchers can explore whether biological pathways known to occur under ideal parameters might exist. Here, it offers a secondary method of investigation beyond clinical studies to determine if a potential mechanism of action produces the same array of patterns clinically and anecdotally recognized by clinicians and patients.

In addition, such a method is not uncommon in other fields, such as toxicology, environmental epidemiology, and neurobiology, where formal Monte Carlo models often precede noteworthy human studies.

Limitations

This study has several important limitations that frame the interpretation of its findings. Foremost, all results derive from a mechanistic Monte Carlo simulation rather than empirical human data. While the model was grounded in established literature on oxidative stress, cannabinoid biology, and hair-cycle regulation, simulated relationships cannot substitute for clinical observation, and the reported associations reflect the internal structure of the model rather than verified causal effects in real populations.

Baseline follicular density and cannabis exposure parameters were derived from independent, publicly available trichoscopy reference intervals and national distribution statistics, which were not originally designed for unified biological modeling. Their integration, therefore, introduces structural uncertainty that cannot be reduced without primary clinical data. In addition, the oxidative-load framework relied on simplified coefficient weighting to approximate the relative contributions of cannabis exposure, sex, and diet. These coefficients do not capture nonlinear biochemical reaction kinetics, gene-environment interactions, or interpersonal variability. Several relevant confounders - including psychosocial stress, micronutrient status, endocrine variation, sleep disruption, and additional environmental toxins - were not explicitly modeled.

Hair-cycle behavior was represented using changes in follicular density and SAHL severity across two timepoints, which does not capture full anagen-catagen-telogen phase dynamics or regional scalp topography. Moreover, the SAHL instrument was modeled to represent population-level symptom diversity, rather than being derived from validated longitudinal psychometric datasets. As no human tissues or biological specimens were analyzed, the model does not account for intrafollicular biochemical gradients, histologic architecture, or direct oxidative biomarkers.

Although simulation-based approaches offer important conceptual value when human data are limited, their outputs must be interpreted cautiously. Controlled clinical investigations, integrating objective exposure quantification, trichoscopy, molecular oxidative biomarkers, and appropriate covariate control, will be required to confirm, refute, or refine the mechanistic patterns generated by this modeling work.

Implications and future directions

Despite these limitations, the present findings suggest that cannabis-related oxidative stress is theoretically capable of producing hair-cycle patterns consistent with early telogen effluvium. This provides a mechanistic basis for further inquiry and may help clinicians contextualize anecdotal patient reports of shedding associated with cannabis use.

Future research should incorporate prospective human cohorts with objective exposure quantification; scalp biospecimens permitting direct measurement of oxidative markers (e.g., 8-OHdG and MDA); high-resolution trichoscopy or optical coherence tomography (OCT) to track hair-cycle transitions in real time; and genetic and molecular assays evaluating CB1 signaling pathways in follicular microenvironments. Simulation-based approaches can also be expanded to incorporate circadian variation, endocrine modulators, or multi-stressor environments, to better reflect the complexity of hair biology.

## Conclusions

This mechanistic Monte Carlo simulation demonstrates that biologically plausible interactions between smoked cannabis exposure, oxidative stress, and hair-cycle disruption are capable of generating patterns that resemble early telogen effluvium. Higher modeled cannabis exposure produced weak-to-moderate increases in symptom severity and decreases in follicular density, consistent with the direction and magnitude of effects reported for other environmental and metabolic stressors in the dermatologic literature. These findings do not represent clinical data and do not imply causality. Instead, they show that oxidative-load mechanisms documented in experimental and biochemical studies can, when applied to a simulated cohort, yield emergent patterns that mirror clinically observed shedding phenotypes.

Such results champion the use of simulation-based research in disciplines where human research is not always feasible due to practical, metric, or ethical constraints. Thus, the ability to suggest that cannabis-induced oxidative stress could potentially (and theoretically) mediate stability of the hair cycle provides a basis for further research. In the end, however, a more definitive clinical study with objective exposures, trichoscopy, and biological measures will need to assess whether such a connection exists in real life, and whether cannabis-induced exposure could be a modifiable risk factor for diffuse shedding, or merely associated with other traumas that affect hair integrity. In summary, to our knowledge, this study represents a first step toward understanding the potential effects of burning-induced oxidative burden on follicular homeostasis. It outlines future studies of cannabinoid biology, redox status, and hair-cycle homeostasis.
